# Sex Differences in a Rat Model of Peripheral Neuropathic Pain and Associated Levels of Endogenous Cannabinoid Ligands

**DOI:** 10.3389/fpain.2021.673638

**Published:** 2021-06-04

**Authors:** Laura Boullon, David P. Finn, Álvaro Llorente-Berzal

**Affiliations:** Department of Pharmacology and Therapeutics, School of Medicine, Centre for Pain Research and Galway Neuroscience Centre, National University of Ireland Galway, Galway, Ireland

**Keywords:** sexual dimorphism, chronic neuropathic pain, spared nerve injury, depression, anxiety, endogenous cannabinoid ligands

## Abstract

Chronic neuropathic pain is a major unmet clinical need affecting 10% of the world population, the majority of whom suffer from co-morbid mood disorders. Sex differences have been reported in pain prevalence, perception and response to analgesics. However, sexual dimorphism in chronic neuropathic pain and the associated neurobiology, are still poorly understood. The lack of efficacy and the adverse effects associated with current pharmacological treatments, further underline the need for new therapeutic targets. The endocannabinoid system (ECS) is a lipid signalling system which regulates a large number of physiological processes, including pain. The aim of this study was to investigate sexual dimorphism in pain-, anxiety- and depression-related behaviours, and concomitant alterations in supraspinal and spinal endocannabinoid levels in the spared nerve injury (SNI) animal model of peripheral neuropathic pain. Sham or SNI surgery was performed in adult male and female Sprague-Dawley rats. Mechanical and cold allodynia was tested weekly using von Frey and acetone drop tests, respectively. Development of depression-related behaviours was analysed using sucrose splash and sucrose preference tests. Locomotor activity and anxiety-related behaviours were assessed with open field and elevated plus maze tests. Levels of endocannabinoid ligands and related *N*-acylethanolamines in supraspinal regions of the descending inhibitory pain pathway, and spinal cord, were analysed 42 days post-surgery. SNI surgery induced allodynia in rats of both sexes. Female-SNI rats exhibited earlier onset and greater sensitivity to cold and mechanical allodynia than their male counterparts. In male rats, SNI induced a significant reduction of rearing, compared to sham controls. Trends for depressive-like behaviours in females and for anxiety-like behaviours in males were observed after SNI surgery but did not reach statistical significance. No concomitant alterations in levels of endogenous cannabinoid ligands and related *N*-acylethanolamines were observed in the regions analysed. Our results demonstrate differential development of SNI-induced nociceptive behaviour between male and female rats suggesting important sexually dimorphic modifications in pain pathways. SNI had no effect on depression- or anxiety-related behaviours in animals of either sex, or on levels of endocannabinoid ligands and related *N*-acylethanolamines across the regions involved in the descending modulation of nociception at the time points investigated.

## Introduction

Pain, as defined by the International Association for the Study of Pain (IASP) is considered “an unpleasant sensory and emotional experience associated with, or resembling that associated with, actual or potential tissue damage” (1). Pain can be classified as acute or chronic, dependent on its duration. Chronic pain, pain persisting over 3 months, can be classified as inflammatory, idiopathic or neuropathic (2).

Neuropathic pain, caused by a lesion or disease affecting the somatosensory system, has a prevalence of 10% in the total world population (3), and is one of the major unmet clinical needs. Despite its higher prevalence in women, females are still underrepresented in pre-clinical studies relevant to chronic neuropathic pain, with <20% of pre-clinical/animal studies including sex as a factor (4). This situation leaves a gap in the understanding of pain neurobiology.

In addition, several clinical reports indicate that chronic pain and mood disorders, such as anxiety and depression, are highly comorbid (5). Higher prevalence of neuropathic pain disorders, tolerance to analgesics, and severe pain perception have been described in patients with anxiety or depression (6). Furthermore, mood disorders have been reported to be more than twice as prevalent in females than males (7–9). Current pharmacotherapies for chronic pain disorders, including antidepressants, anticonvulsants, local anaesthetics, non-steroidal anti-inflammatory drugs, or opioids, are limited in terms of their efficacy and adverse effect profiles (10, 11). Thus, there is a need to identify new analgesic targets.

The endogenous cannabinoid system (ECS) is a complex signalling system comprised of cannabinoid type 1 (CB_1_) and cannabinoid type 2 (CB_2_) receptors; endocannabinoid ligands: anandamide (AEA) and 2-arachidonoylglycerol; and catabolizing enzymes: fatty acid amide hydrolase (FAAH) and monoacylglycerol lipase (MAGL). Several other related biogenic lipids, including *N*-acylethanolamines: oleoylethanolamine (OEA) and palmitoylethanolamine (PEA), are involved in the regulation of common endocannabinoid-mediated phenomena (12–14) and thus are recognised as endocannabinoid-related compounds. The ECS is involved in numerous physiological processes, including memory, mood and pain (15, 16). The signalling machinery of the ECS is expressed at neuronal synapses throughout the pain circuitry and regulates nociceptive processing and perception (17). Activation of cannabinoid receptors on presynaptic nerve terminals inhibits neurotransmission, resulting in antinociception. The majority of cannabinoid receptors expressed within the central nervous system, are type 1 (CB_1_) and localised in dorsal horn and supraspinal regions involved in the descending inhibition of nociception: prefrontal cortex (PFC), periaqueductal grey (PAG), amygdala, and rostral ventromedial medulla (RVM); which also play important roles in anxiety and depression circuitries (18, 19).

Sexual dimorphism has been demonstrated in the ECS, from pre-clinical animal models to humans. Sex differences have been described in the expression and activity of CB_1_ receptors in PFC and amygdala (20, 21). Sex-dependent efficacy of phytocannabinoid- and CB_2_-mediated nociception after nerve injury has been found in humans (22, 23) and animal models of neuropathic pain (24–27). Differences in the density and affinity of brain endocannabinoid receptors and ligands have also been observed at different stages of the hormonal cycle (28, 29). It is possible therefore, that sex-dependant alterations within the ECS may contribute to sexual dimorphism in neuropathic pain.

The aim of this study was to investigate sexual dimorphism in pain-, anxiety- and depression-related behaviours, and concomitant alterations in supraspinal and spinal endocannabinoid levels in the spared nerve injury (SNI) animal model of peripheral neuropathic pain.

## Materials and Methods

### Animals

Adult (8–9 weeks old) male (~250 g) and female (~200 g) Sprague-Dawley rats were purchased from Charles River UK (Margate, United Kingdom). Animals were pair-housed with water and food (14% Harlan Teklad 2014 Maintenance Diet, Envigo, Huntingdon, Cambridgeshire, United Kingdom) available *ad libitum*. Animal holding room was maintained at a constant temperature of 21 ± 2°C and intervals of 45–55% of humidity. All the procedures were performed during the light phase (8:00–20:00) of the standard lighting conditions 12:12 h.

The experimental procedures were approved by the Animal Care and Research Ethics Committee, National University of Ireland Galway. The present experiment was performed in accordance with the ARRIVE guidelines (30); under licence from the Health Products Regulatory Authority in the Republic of Ireland and in accordance with EU Directive 2010/63.

### Surgery

Animals were randomly assigned to surgery groups, Sham or SNI, according to sex (*n* = 10/group, 4 experimental groups). A vaginal swab for the investigation of the oestrus cycle stage was taken from Sham/SNI female groups prior to the surgery. Peripheral sensitisation was induced using the Spare Nerve Injury surgery (SNI), described by Decostered and Woolf (31). The SNI surgery comprises of ligation and axotomy of two of the three branches of the sciatic nerve: the common peroneal and tibial nerves, keeping the third branch, the sural nerve, completely intact. The fur was shaved on the lateral surface of the left hind paw and the area was disinfected with iodine solution ensuring aseptic conditions. A longitudinal incision following the femoral line was made directly through the biceps, exposing mentioned branches of the sciatic nerve. The common peroneal and the tibial nerves were ligated and transected with two different knots at opposite ends of the stump, using 5-0 mersilk sutures. Individual sutures were used to close the muscle incision while intradermal sutures were used to close the skin, avoiding the reopening of the wound by biting. Anaesthesia was monitored and maintained using 2–4% isoflurane in 0.8 L/min O_2_ during the surgical procedure. Respiratory rate and pedal withdrawal reflexes were also monitored every 5 min. After the surgical procedure, rats were singly housed and monitored during a recovery period of 30 min before returning them to their home cages. Enrofloxacin (10 mg/kg) was administrated via subcutaneous (s.c.) injection once a day, prior to surgery and for 5 consecutive days post-surgery, to prevent post-surgical infection. Body weight was monitored once a week. General health and well-being were monitored daily during the total time of the experiment.

### Behavioural Testing

All behavioural tests were conducted at least 7 days post-surgery. Each test was consistently performed in the same room under similar environment conditions. EthoVision XT software (Noldus Information Technology, Wageningen, The Netherlands), which allows for continuous event recording and posterior scoring, was used for the analysis of Sucrose Splash test (SS), Open Field test (OF) and Elevated Plus Maze test (EPM). A trained researcher, blind to the experimental conditions, rated sucrose consumption in sucrose preference test, depression-related behaviour in the SS test and anxiety-related behaviours in the OF and EPM tests.

#### Mechanical Allodynia

Von Frey testing was performed to assess mechanical allodynia 7, 14, 21, 28, and 42 days after surgery. Rats were placed in an elevated metal wire grid, divided into six individual compartments (14 × 20 × 25 cm^3^), permitting the stimulation of the lateral plantar surface of the hind paw. Animals were habituated to the arena for 15 min prior to testing. The test was performed following the up-and-down method described by Dixon (32). Each animal received a maximum of 9 nylon von Frey filament stimulations (Touch Test Sensory Evaluator 58011, Stoelting, IL, USA) per hind paw, starting with the 2 g filament. Each filament was applied once, perpendicular to the lateral plantar surface of the hind paw, the area innervated by the intact sural nerve. The mechanical withdrawal threshold (g) for each paw was calculated using the formula: 10^[log(last filament)+k*0.3]^, where the constant k was determined by the response-pattern (32).

#### Cold Allodynia

Acetone Drop testing was performed to assess cold allodynia. Animals rested in the arena for 10 min after the von Frey test. The test was performed following the method described by Choi et al. (33) The animals received a total of 3 acetone stimulations per hind paw. A drop of acetone was applied to the lateral plantar surface of the contralateral or ipsilateral hind paw, using a blunt needle connected to a 1 ml syringe, avoiding touching the skin. During the 1 min stimulation, the latency to the first response and the total number of responses were scored.

#### Depression-Related Behaviours

##### Sucrose Splash Test

The sucrose splash (SS) test was performed to assess self-grooming behaviour, which is an ethologically relevant behaviour in rodents (34). A decrease in self-grooming behaviour is indicative of reduced self-care and motivation in rodents, and potentially reflective of anhedonia-like behaviour (35). At post-surgery day 34, animals were placed in a clear Plexiglass box (30 × 30 × 40 cm^3^) with 30 lux light conditions for 10 min habituation period. After the initial habituation to the arena, 10% sucrose solution was squirted twice, using a spray bottle, on the dorsal fur of the rat. The animal was immediately returned to the arena and observed for another 10 min. A video camera located underneath the arena recorded the test session permitting the scoring of frequency and latency to grooming during the sucrose splash test.

##### Sucrose Preference Test

The sucrose preference (SP) test assessed the development of anhedonia, categorised as a measure of depression-related behaviours (36). Animals were trained for 4 days prior to testing (37). The sucrose preference test was performed in home-cages with no additional testing occurring during this period. Each home cage was fitted with space for two drinking bottles in opposite sides (right-left). During the first and second day of training, rats were allowed to drink 1% sucrose solution, instead of water, *ad libitum* during 24 h. During the third and fourth day of training, rats were allowed to drink water *ad libitum* for 24 h. The position of the drinking bottle changed from the right to the left on the second and fourth training days. The training period acclimatised the rats to drink from both sides of the cage. On the test day, at post-surgery day 28, a bottle with 1% sucrose solution was fitted on the right position, and a bottle with water was fitted on the left position. After 12 h, the position of the drinking bottles was swapped to avoid side preference. The test finished 12 h after the swap when bottles were removed, and animals returned to their drinking regime. During the training and test periods, bottles were weighted before and after any human manipulation. The preference for sucrose solution was calculated and presented as $\%\;Sucrose\;Preference=\frac{Sucrose\;intake}{Total\;intake}\;\times \;100$.

#### Anxiety-Related Behaviours

##### Open Field Test

The Open Field (OF) test was performed for the assessment of locomotor activity and anxiety-related behaviours. Significant reductions in the distance moved and velocity were considered possible alterations in the locomotor activity due to spared nerve injury. In addition, a decrease in frequency or time of rearing activity, or a decrease in the number of entries and duration in the centre zone (45 cm of diameter), were assessed as deficits of exploratory behaviour thus indicative of anxiety-related behaviours (38). The test was performed at post-surgery day 35 in a circular arena consisted of a reflective aluminium floor of 75 cm diameter and reflective aluminium wall of 40 cm height. Animals were placed in the centre of the novel open environment, illuminated with 100 lux. A video camera positioned above the floor of the arena was used to record the behaviours for 5 min, which were scored later on using EthoVision XT.

##### Elevated Plus Maze Test

The Elevated Plus Maze (EPM) test was performed to assess anxiety-related behaviours, based on the natural avoidance of rodents to height and open spaces (39). The frequency and number of entries into the open arms is thought to be indicative of anxiety-like behaviours (39). The arena was elevated 50 cm above the floor of the testing room and consisted of a white plus-shaped wooden maze with two arms enclosed by walls (30 cm high) and two open arms with no enclosure. Each arm was 50 cm in length and 10 cm in width. A central platform (10 × 10cm^2^) connected the four arms. The light levels were fixed at 45 lux in the open arms and 15 lux in the closed arms. Immediately after the exposure to OF, rats were placed in the centre zone of the EPM arena with the head facing the open arm and allowed to explore for 5 min. The behaviours were recorded with a video camera located above the arena and scored later on using EthoVision XT.

### Tissue Collection

Animals were euthanized by live decapitation on post-surgery day 42, after mechanical and cold allodynia behavioural testing. A vaginal swab for the investigation of the oestrus cycle stage was taken from female rats post-euthanasia. The following brain regions: prefrontal cortex (PFC), periaqueductal grey (PAG), ipsilateral/contralateral amygdala and rostro ventromedial medulla (RVM) were gross-dissected on ice-cold metal dissection plates and snap-frozen for post-mortem analysis. Spinal cord was extracted by laminectomy; the dorsal lumbar area was differentiated in ipsilateral and contralateral, and snap-frozen on dry ice for later analysis.

### Measurement of Endocannabinoids and Related N-acylethanolamines by Liquid Chromatography Coupled to Tandem Mass Spectrometry (LC-MS/MS)

Quantification of endocannabinoids (AEA, 2-AG) and related *N*-acylethanolamines (PEA, OEA) levels in brain and spinal cord tissue was carried out following a lipid extraction method described previously (40, 41). Two hundred microlitres of 100% acetonitrile containing deuterated internal standard for endogenous cannabinoid ligands (2.5 ng d8-AEA, 50 ng d8-2-AG, 2.5 ng d4-PEA, 2.5ng d4-OEA; Cayman Chemicals, Biosciences, UK) and 75 μl of 100% pure acetonitrile were added to the samples. The tissue was homogenised for ~6 s using an ultrasonic homogeniser (Mason, Dublin, Ireland). Immediately after homogenization, samples were centrifuged at 14,000 × g for 15 min at 4°C (Hettich^®^ centrifuge Mikro 22R, Hettich, Germany). Samples were immediately placed on ice. A standard curve 1/4 dilution was prepared where the highest standard (Standard 10) was made up by adding 25 μl of 100% acetonitrile containing a known fixed amount of non-deuterated internal standard (25 ng AEA, PEA, OEA and 250 ng 2-AG) to 75 μl of 100% pure acetonitrile solution. Finally, 200 μl of 100% acetonitrile containing known fixed amount of deuterated internal standard was added to each standard. Forty microlitres of each sample and standard curve point were added to HPLC vials.

Mobile phases comprised of Solution A (HPLC-grade water with 0.1% (v/v) formic acid) and Solution B (100% acetonitrile with 0.1% (v/v) formic acid) with a flow rate of 0.2 ml/min, onto a Waters Atlantis T3 HPLC column (3 μm particle size dimension, 100 mm length, 2.1 mm diameter; Waters, UK). Reversed-phase gradient elution was initiated at 2% acetonitrile for the first 3 min, set to 65% acetonitrile at 3.1 min for 1 min and then ramped linearly up to 100% acetonitrile at 8 min and held at 100% acetonitrile until 16 min. At 16.1 min, the gradient returned to initial conditions for a further 12 min re-equilibrating the column before the next injection. Analyte detection was carried out in electrospray-positive ionisation mode on an Agilent 1100 HPLC system coupled to a triple quadrupole 6460 mass spectrometer (Agilent Technologies, Cork, Ireland). Ratiometric quantification was calculated using Agilent Mass-Hunter Quantitative Analysis Software (Agilent Technologies, Cork, Ireland). The amount of analyte in unknown samples was calculated from the analyte/internal standard peak area response ratio using a 10-point calibration curve constructed from a range of concentrations of the non-deuterated form of each analyte and a fixed amount of deuterated internal standard.

### Statistical Analysis

IBM SPSS Statistics 26.0 statistical software (Chicago, USA) was used for the statistical analysis of data. Normality and homogeneity were assessed by Kolmogorov-Smirnov comparisons and Levene's test, respectively. Parametric statistical analysis was performed in normally distributed datasets, using two-way ANOVA (factors: sex and surgery) followed by Tukey HSD (Honest Significant Difference) *post-hoc* test for pairwise comparisons. Three-way ANOVA (factors: sex, surgery and side) was used to analyse levels of endogenous cannabinoid ligands in lateralised regions of the central nervous system: amygdala and spinal cord. Non-parametric datasets were analysed using Kruskal-Wallis test followed by Mann-Whitney *U post-hoc* test with Bonferroni-Holm corrections. The influence of oestrus cycle on mechanical and cold allodynia, was analysed as categorical binary variable (high-low) using a binomial logistic regression with surgery and stage of the oestrus cycle as independent variables. All data are expressed as group means ± standard error of the mean (± SEM) for presentation/readability purposes and considered significant when *p* < 0.05.

## Results

### Spared Nerve Injury Induced Sexual-Dimorphic Development of Mechanical Allodynia in Sprague-Dawley Rats

**Figure 1:**
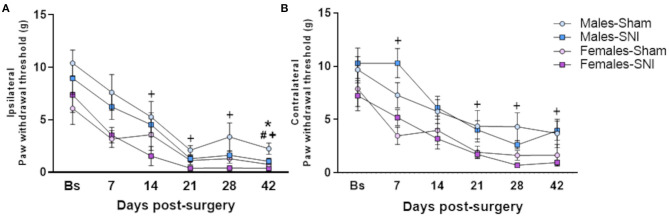
Von Frey test. Investigation of mechanical allodynia in male and female rats following Sham or Spared Nerve Injury surgery. Ipsilateral **(A)** and contralateral **(B)** paw withdrawal thresholds at the following time points: baseline pre-surgery (Bs), post-surgery days 7, 14, 21, 28, and 42. Data expressed as mean ± SEM (*n* = 10 per group). Kruskal Wallis test followed by Mann-Whitney *U post-hoc* test with Bonferroni-Holm corrections (*P* < 0.05) *Males-Sham vs. Males-SNI, # Females-Sham vs. Female-SNI, + Males-SNI vs. Females-SNI.

Significant differences between experimental groups were observed for mechanical allodynia, measured as paw withdrawal thresholds (PWT), of the ipsilateral hind paw (Figure 1A), at all the time-points tested [Kruskal-Wallis: PSD7: H(3) = 8.161, *p* < 0.01; PSD14: H(3)=11.019, *p* < 0.05; PSD21: H(3) = 12.110, p <0.01; PSD28: H(3) = 12.057, *p* < 0.01; PSD42: H(3) = 20.199, *p* < 0.001]. *Post-hoc* analysis revealed that SNI induced a robust decrease of PWT in the ipsilateral hind paw of male and female animals at post-surgery day 42 (Figure 1A). Interestingly, female-SNI rats showed lower ipsilateral PWT compared to their male counterparts from post-surgery day 14 (Figure 1A). This sex-dependent effect on mechanical hypersensitivity was also observed in the contralateral PWT of SNI rats (Figure 1B), however, no significant reduction of the contralateral PWT was observed in SNI groups compared to their Sham counterparts (Figure 1B). No significant effect of the oestrus cycle was observed within female experimental groups.

### Spared Nerve Injury Induced Sexual-Dimorphic Development of Cold Allodynia in Sprague-Dawley Rats

**Figure 2:**
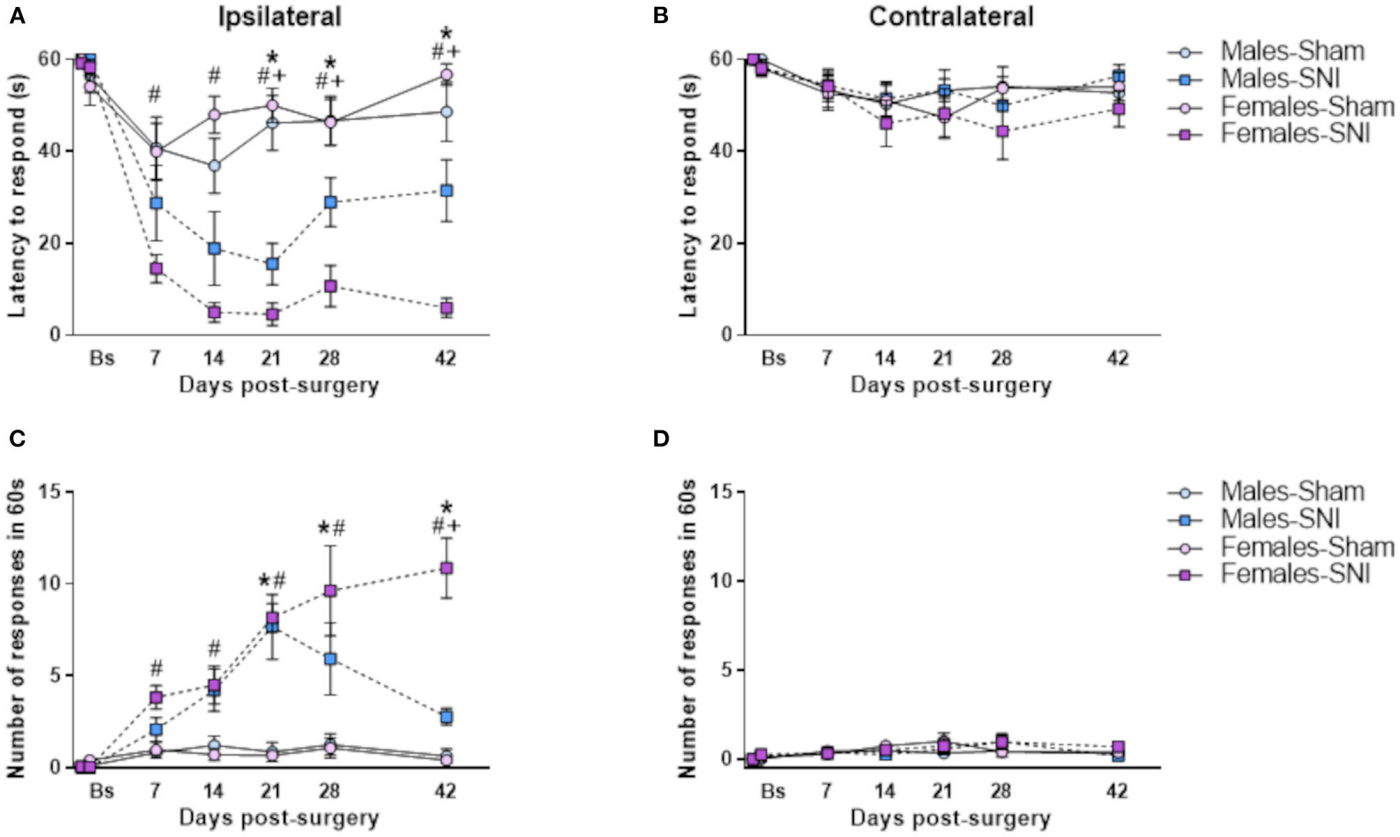
Acetone drop test. Investigation of cold allodynia in male and female rats following Sham or Spared Nerve Injury surgery. Ipsilateral **(A)** and contralateral **(B)** latency to the first response after acetone stimulation; number of responses in 60 s after acetone stimulation of the ipsilateral **(C)** and contralateral **(D)** hind paw, represented at the following time points: baseline pre-surgery (Bs), post-surgery days 7, 14, 21, 28, and 42. Data expressed as mean ± SEM (*n* = 10 per group). Kruskal Wallis followed by Mann-Whitney *U post-hoc* test with Bonferroni-Holm corrections (*P* < 0.05) *Males-Sham vs. Males-SNI, # Females-Sham vs. Female-SNI, + Males-SNI vs. Females-SNI.

The analysis of the latency to first response of the ipsilateral hind paw in the acetone drop test revealed significant differences between experimental groups at all time-points tested [Kruskal-Wallis: PSD7: H(3) = 9.788, *p* < 0.05; PSD14: H(3) = 19.185, *p* < 0.001; PSD21: H(3) = 23.621, *p* < 0.001; PSD28: H(3) = 17.213, *p* < 0.001; PSD42: H(3) = 23.698, *p* < 0.001]. The data show a reduction of the latency to the first response in male- and female-SNI animals, compared to Sham counterparts. In addition, we observed a robust reduction of the latency to the first response in female-SNI compared to male-SNI groups from PSD21 (Figure 2A), suggesting higher nociceptive sensitivity in female compared to male animals following SNI. No significant differences were observed in following analysis of data for the contralateral hind paw (Figure 2B).

Significant differences between experimental groups were also observed in the number of responses to acetone stimulation in the ipsilateral hind paw of male and female rats [Kruskal-Wallis: PSD7: H(3) = 14.007, *p* < 0.001; PSD14: H(3) = 14.108, *p* < 0.01; PSD21: H(3) = 24.448, *p* < 0.001; PSD28: H(3) = 19.381, *p* < 0.001; PSD42: H(3)=29.058, *p* < 0.001]. Further analysis revealed an increase of this parameter in SNI male and female groups (Figure 2C). In addition, an increased number of responses in female-SNI rats was observed at PSD7, an effect not observed in male-SNI groups until post-surgery day PSD21 (Figure 2C), suggesting earlier onset of cold allodynia in female-SNI groups. As previously observed, female-SNI rats exhibited a higher number of responses than male-SNI rats from PSD21. No significant differences were observed in the contralateral hind paw (Figure 2D).

The oestrus cycle did not alter the latency to the first response or the frequency of responses within the female groups.

### Spared Nerve Injury Did Not Induce Depression-Related Behaviours in Sprague-Dawley Rats of Either Sex

**Figure 3:**
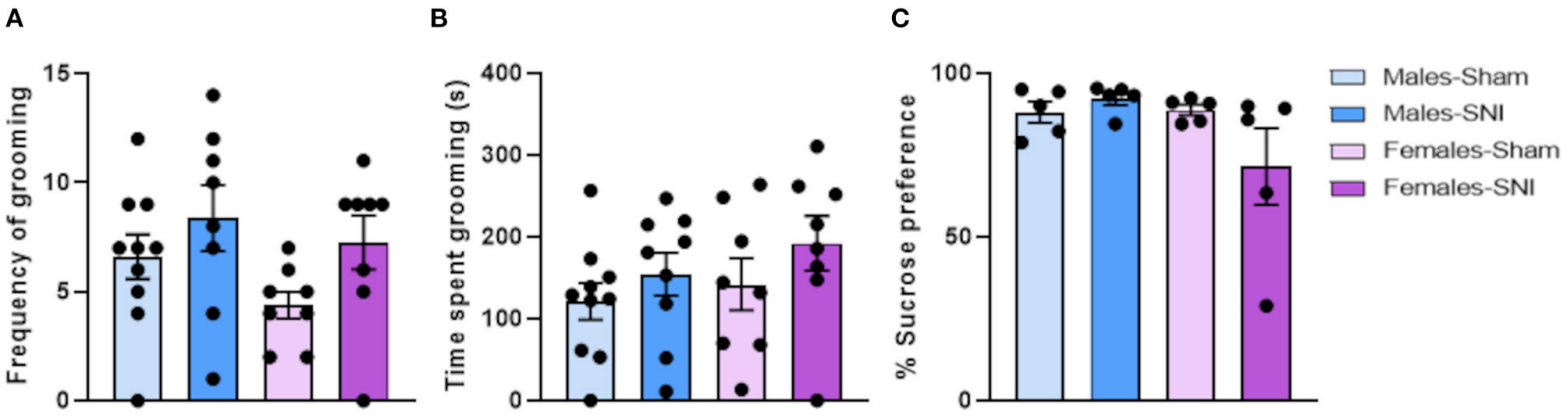
Sucrose Splash and Sucrose preference tests. Investigation of depression-related behaviours following Sham or Spared Nerve Injury surgery in male and female rats. Frequency of grooming **(A)** and cumulative time of grooming (s) **(B)** after sucrose splash exposure, at post-surgery day 28. All the behaviours were analysed during the first 10 min of trial. Percentage of sucrose consumption **(C)** at post-surgery day 34. Percentage of sucrose preference was calculated in each pair-housed cage as: [sucrose intake (g)/total intake (g)] × 100 (see section Sucrose preference Test in Material and Methods for further information). Data expressed as mean ± SEM (sucrose splash test: *n* = 10 per group; sucrose preference test: *n* = 5 per group).

No main effects were observed in the frequency (Figure 3A) or duration (Figure 3B) of grooming behaviour, during sucrose splash test. Moreover, preference for 1% sucrose solution did not differ between groups on post-surgery day 34 (Figure 3C).

### Spared Nerve Injury Did Not Induce Anxiety-Related Behaviours but Decreased Vertical Activity in Male Sprague-Dawley Rats

**Figure 4:**
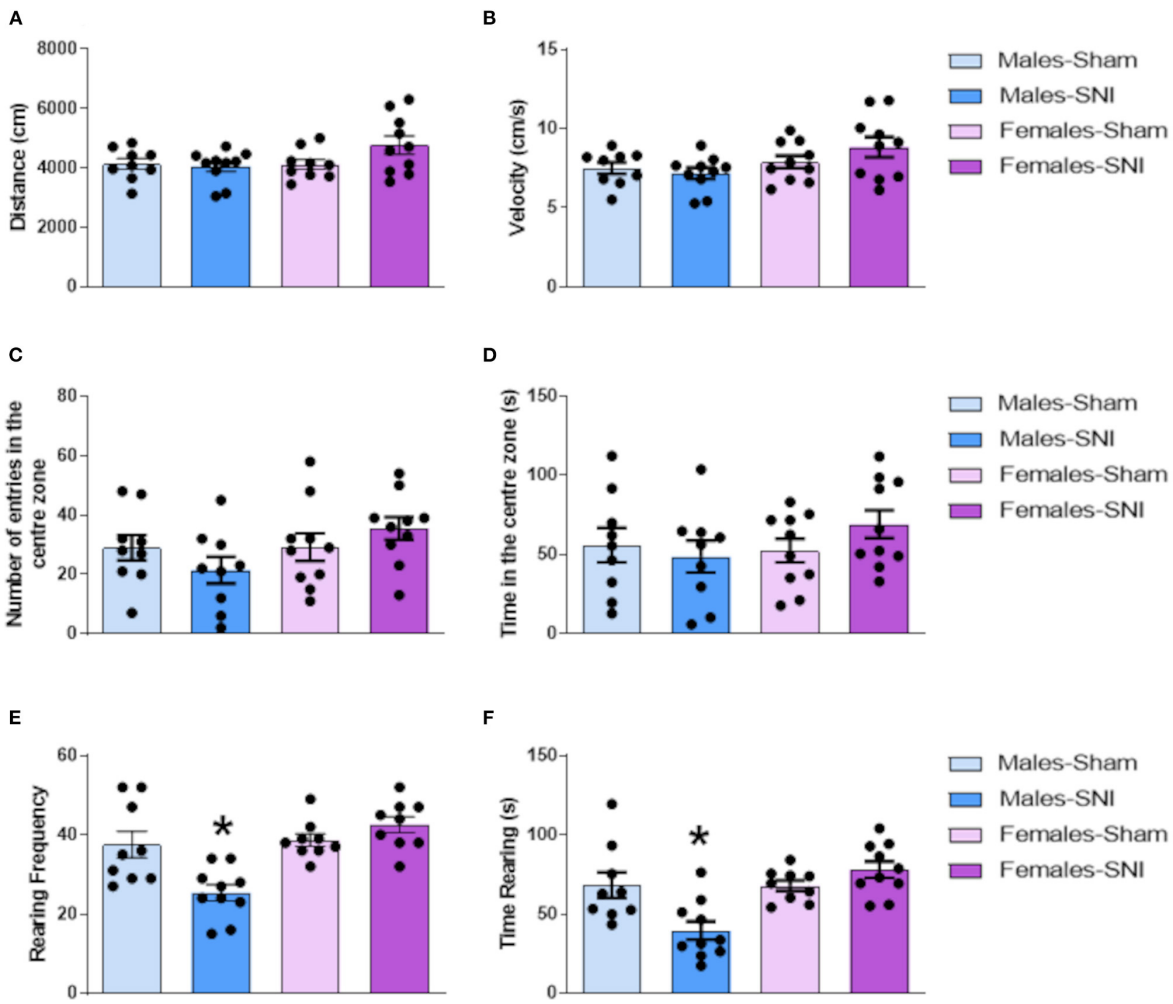
Open Field test. Investigation of locomotor activity and anxiety-related behaviours in male and female rats following Sham or Spared Nerve Injury surgery. Distance moved (cm) **(A)**, velocity (cm/s) **(B)**, number of entries in the centre zone of the arena **(C)**, duration in the centre zone (s) **(D)**, frequency of rearing **(E)**, duration of rearing (s) **(F)** at post-surgery day 35. All the behaviours were analysed during the 5 min trial. Data expressed as mean ± SEM (*n* = 10 per group). Two-way ANOVA followed by Tukey HSD *post-hoc* (*P* < 0.05). *Males-Sham vs. Males-SNI.

In the open field, no main effects were observed in the horizontal locomotor activity, distance moved (Figure 4A) and velocity (Figure 4B), or in the anxiety-related behaviours, number of entries (Figure 4C) and time (Figure 4D) in the centre zone. Interestingly, vertical activity analysis revealed significant main effects of sex [rearing frequency: *F*_(1,34)_ = 15.490; *p* < 0.001); and duration: *F*_(1,34)_ = 10.660; *p* < 0.05)] and sex^*^surgery interactions [rearing frequency: *F*_(1,34)_ = 12.359; *p* < 0.001; and duration: *F*_(1,34)_ = 10.933; *p* < 0.01]. Further analysis revealed lower vertical activity in male-SNI rats compared with Sham counterparts (Figures 4E,F), an effect that was not observed in female animals.

**Figure 5:**
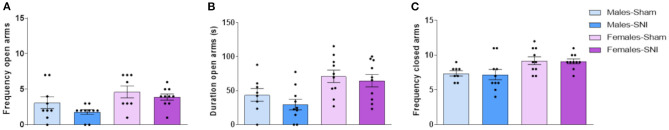
Elevated plus maze. Investigation of anxiety-related behaviours in male and female rats following Sham and Spared Nerve Injury surgery. Frequency of open arms exploration **(A)**, duration of open arms exploration (s) **(B)**, frequency of closed arms exploration **(C)** at post-surgery day 35. All the behaviours were analysed during the 5 min trial. Data expressed as mean ± SEM (*n* = 10 rats per group).

A significant main effect of sex was observed in the frequency of entries [*F*_(1,37)_ = 9.312; *p* < 0.05] and duration [*F*_(1,37)_ = 7.345; *p* < 0.05] in the open arms of the elevated plus maze without altering the frequency of entries in closed arms (Figure 5). Although male-SNI rats exhibited a trend for reduced frequency and duration in the open arms, *post-hoc* analyses did not reveal any significant differences.

### Spared Nerve Injury Did Not Alter Endogenous Cannabinoid Ligand Levels Within Regions Involved in the Descending Modulation of Nociception in Male or Female Sprague-Dawley Rats

**Figure 6:**
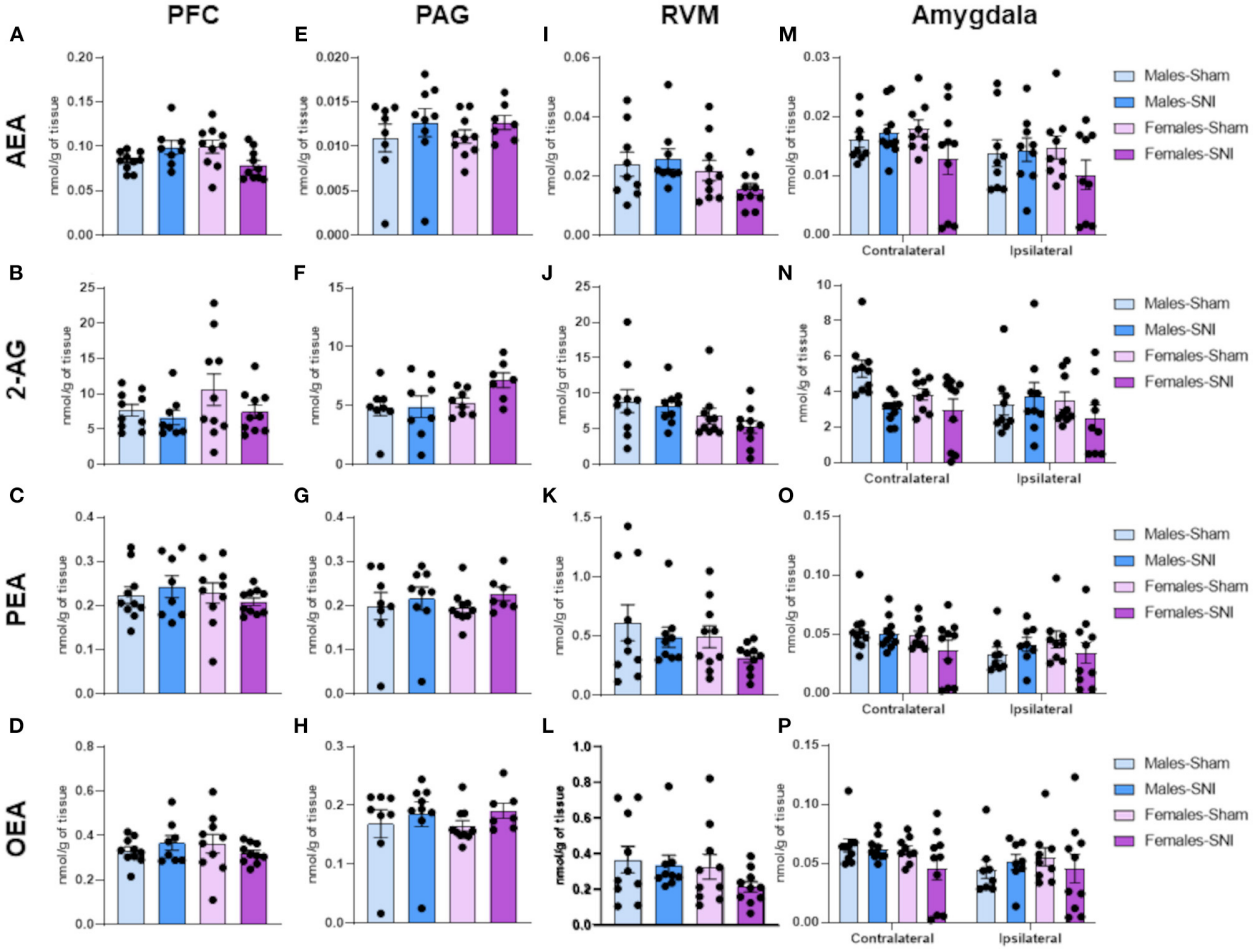
Investigation of endocannabinoid ligand and *N*-acylethanolamine levels (nmol/g of tissue) in male and female rats on day 42 following Sham and Spared Nerve Injury surgery. Levels of AEA, 2-AG, PEA and OEA within brain regions implicated in the modulation of the nociceptive response in the descending pain pathway: prefrontal cortex (PFC; **A–D**), periaqueductal grey substance (PAG; **E–H**); rostral ventromedial medulla (RVM; **I–L**) and contralateral and ipsilateral amygdala **(M–P)**. Data expressed as mean ± SEM (*n* = 10 rats per group).

Statistical analysis revealed no main effects on the levels of endocannabinoids (AEA and 2-AG) and related *N*-acylethanolamines (PEA and OEA) in the PFC (Figures 6A–D), PAG (Figures 6E–H), or RVM (Figures 6I–L). In the amygdala (Figures 6M–P), a main effect of side was observed [*F*_(1,64)_=7.247; *p* < 0.01] for 2-AG (Figure 6N), but subsequent *post-hoc* analysis did not reveal any significant between-group differences.

**Figure 7:**
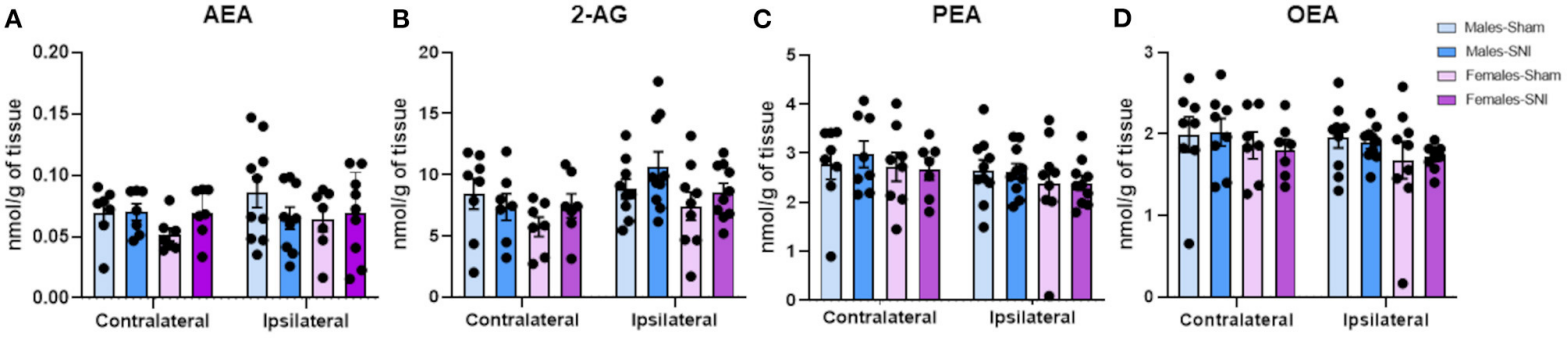
Investigation of endocannabinoid ligand and *N*-acylethanolamine levels (nmol/g of tissue) in dorsal lumbar spinal cord of male and female rats on day 42 following Sham and Spared Nerve Injury surgery. Levels of AEA **(A)**, 2-AG **(B)**, PEA **(C)** and OEA **(D)** were measured by liquid chromatography coupled to tandem mass spectrometry. Data expressed as mean ± SEM (*n* = 10 rats per group).

Spinal cord levels of AEA, 2-AG, PEA or OEA did not differ between groups (Figure 7).

## Discussion

Despite the high incidence of neuropathic pain in the world population, the neurobiology underlying chronic neuropathic pain is still poorly understood. The higher incidence of neuropathies in women compared to men, focus the attention on sex as a crucial factor for the understanding of the mechanisms associated with this disease. Nevertheless, more than 80% of pre-clinical investigations exclude females as subjects, leaving “sex” out of the pain research equation (4).

The study presented herein compares long-term development of mechanical and cold allodynia, following peripheral nerve injury, between sexes. The development of these nociceptive behaviours is common in animal models of neuropathic pain (42, 43) and are well-known features of neuropathic pain symptomatology in patients following peripheral nerve damage, independently of the origin of the lesion (44, 45). Spared Nerve Injury induced mechanical and cold allodynia in adult male and female Sprague-Dawley rats. Decreases in the ipsilateral paw withdrawal threshold, for mechanical allodynia, was progressively observed from post-surgery day 7. Unexpectedly, a delay in the development of significant mechanical allodynia was observed in rats of both sexes. Use of antibiotics immediately after the surgery may be responsible for this altered nociceptive perception through selective actions and disruption of gut bacteria (46). Several animal models with altered motility and nociceptive perception, have provided evidence of gut microbiota and enteric neurons communication (47). The enteric nervous system plays a key role in nociceptive processes, as it comprises primary afferent neurons projecting to the dorsal horn in the spinal cord (48). Therefore, administration of enrofloxacin (5 mg/kg) to avoid infections during the surgical procedure, and the following 5 days, may be contributing to the observed delay in the development of allodynia. Nevertheless, robust sensitivity to mechanical stimuli was described in male- and female-SNI rats at day 42 post-surgery.

Changes were also observed in sham sensitivity to mechanical stimulation compared to baseline; an effect previously reported in sham controls for different peripheral nerve injury models (49–51). Changes observed in Sham groups may be related to the possible sensitisation of the hind paws to testing over time. As previously described, repetitive low-intensity stimulation with von Frey filaments of the hind paw in non-operated rats was associated with progressive decrease in mechanical withdrawal thresholds (52).

A robust cold allodynia response was detected in female-SNI groups 1 week after surgery, an effect not observed in male-SNI groups; suggesting an earlier onset of this pain-related behaviour in female rats. The sensitivity to mechanical and cold allodynia was significantly greater in male- and female-SNI rats compared to their Sham controls, confirming that peripheral nerve injury induced the expected pain-related phenotype. Furthermore, female-SNI groups not only experienced an earlier onset but a greater sensitivity to mechanical and cold stimuli than their corresponding male counterparts, supporting the presence of sexual dimorphism in nociceptive behaviour. Studies on sex differences during nociceptive processing have focused their attention on receptor and ion channel alterations at neural membranes, disruption of nociceptive signal transmission within the nervous system or pathological synapse formation between neighbouring neurons (53). In addition, the molecular and behavioural variations induced by the oestrous cycle has progressed from being considered as a reason for not using female rodents in research, to focus the attention for the better understanding of sexual dimorphism in the field of pain research (54–56). In fact, it has been demonstrated that variations between stages of the oestrus cycle can affect nociceptive perception in females (55, 56). In the present study, a binomial logistic regression was used to analyse if the stage of the oestrus cycle was a predictor of the nociceptive behaviour exhibited by females. However, no significant correlation was found, suggesting that the stage of the oestrus cycle did not influence pain-related behaviours in the current study.

SNI has been proven useful to model co-morbid mood disorders that have been described in more than the 50% of patients suffering from chronic pain (57–61). A recent study from Wang and colleagues has demonstrated long-term SNI-induced depression-related behaviours (14 and 56 days following SNI surgery) in the sucrose preference and the forced swim tests (62). In the current experiment, SNI did not induce depression-related behaviours in either male or female animals 28 and 34 days after the surgery. A non-significant reduction of the preference for sucrose consumption was observed in female-SNI groups (Figure 3A), although further investigation at later time points may be warranted.

Impairments of locomotor activity were not observed in either male or female rats following SNI surgery on the ipsilateral limb. Animal weight was a factor considered during the investigation of this parameter, with no further implication in either sex.

SNI altered exploratory behaviour during the open field test. Male-SNI groups exhibited a significant reduction of frequency and duration of rearing activity, compared to Sham control groups. This effect was not observed in female-SNI rats, suggesting differential effects of SNI on this pain-related behaviour between the sexes.

SNI did not affect the performance of either male or female rats in the elevated plus maze test. Some pre-clinical studies have observed anxiety-related behaviours following peripheral neuropathy, with the majority only showing positive manifestations at least 4 weeks after neuropathy induction (43, 63, 64). The analyses of depression- and anxiety-associated behaviours, within this study, were conducted at early post-neuropathy induction stages in which nociceptive chronicity may not yet be well-established. This finding corroborates the importance of time for the detection of emotional disorders following chronic pain, previously observed in the literature (15, 43, 65).

The expression of the ECS throughout the descending pain pathway (66) and its role in the modulation of glutamatergic and GABAergic neurotransmission during nociceptive processing (67) is well-established. Activation of CB_1_ receptors activates the descending inhibitory pathway through the inhibition of GABA release in the periaqueductal grey and rostral ventromedial medulla (17, 68), which bidirectionally modulate spinal cord dorsal horn excitability for nociceptive transmission. Despite the reported evidence of the ECS in the modulation of nociceptive processing (66, 69) and the presence of sex differences within the ECS (23, 70), we did not observe changes in the levels of endocannabinoid ligands in the descending pain pathway. However, our results are not sufficient to discard the possibility of alterations in endocannabinoid tone at other time points following SNI-surgery. Evidence suggests that longer periods post-SNI induction may be needed for the establishment of endocannabinoid alterations associated with a more robust nociceptive phenotype (71). For instance, Petrosino et al. (72) only observed an increase of AEA and 2-AG levels in the PAG, RVM and spinal cord following chronic constriction injury in male animals, when they expressed high levels of hyperalgesia and mechanical allodynia (72). Considering our behavioural results, it is conceivable that a later time point is required to observe the highest levels of mechanical and cold allodynia and associated increases in AEA and 2-AG levels.

It is also possible, that chronic neuropathic pain induced alterations of the levels of endocannabinoid ligands in specific small sub-nuclei of the descending pain pathway. It has been previously observed that subregions of the same brain area can play distinctive roles in pain neurotransmission and related emotional modulation (18). However, the use of gross-dissected tissue would not have allowed us to observe these subnuclei-dependent effects. Furthermore, as different models of chronic pain have shown, the antinociceptive effects of the endocannabinoid system seem to rely on the activation of both CB_1_ and CB_2_ receptors in females, but only on the activation of CB_1_ receptor in males (70) highlighting the importance of receptor mechanisms in addition to considerations of endogenous ligand levels.

In conclusion, the current study describes sexual dimorphism in the development of mechanical and cold allodynia following peripheral nerve injury. Females exhibited an earlier onset and a greater sensitivity to mechanical and cold allodynia than males, following SNI; suggesting sex differences in the mechanisms underlying nociceptive processing. These results, together with the clinical evidence of a higher incidence of neuropathies in women compared to men, further point to the need to consider “sex” as a crucial factor to improve the understanding of pain neurobiology. Although the sex differences in nociceptive perception observed were not associated with alterations in the levels of endogenous cannabinoid ligands in spinal cord or the supraspinal regions involved in the descending inhibitory pain pathway at the time points tested, further studies are required to investigate the plasticity and potential involvement of the ECS in the sexual dimorphism associated with peripheral neuropathy over longer periods post-injury.

## Data Availability Statement

The raw data supporting the conclusions of this article will be made available by the authors, without undue reservation.

## Ethics Statement

The animal study was reviewed and approved by Animal Care Research Ethics Committee of the National University or Ireland, Galway and the Health Products Regulatory Authority in the Republic of Ireland.

## Author Contributions

DF and ÁL-B developed the experimental design, reviewed, and edited the manuscript. LB and ÁL-B carried out the *in vivo* study and related data analysis. LB carried the *ex vivo* assays, related data analysis and wrote the first draught of the manuscript. All authors contributed to the article and approved the submitted version.

## Conflict of Interest

The authors declare that the research was conducted in the absence of any commercial or financial relationships that could be construed as a potential conflict of interest.
